# Assessment of Common Hematologic Parameters and Novel Hematologic Ratios for Predicting Piroplasmosis Infection in Horses

**DOI:** 10.3390/ani15101485

**Published:** 2025-05-20

**Authors:** Juan Duaso, Alejandro Perez-Ecija, Esther Martínez, Ana Navarro, Adelaida De Las Heras, Francisco J. Mendoza

**Affiliations:** 1 Department of Animal Medicine and Surgery, University of Cordoba, 14014 Cordoba, Spain; juanduaso@gmail.com (J.D.); v02hesaa@uco.es (A.D.L.H.); fjmendoza@uco.es (F.J.M.); 2 Gasset Laboratory, DAV Salud Group SL, 18200 Granada, Spain; esther@grupodavsalud.es (E.M.); ana@grupodavsalud.es (A.N.)

**Keywords:** *Babesia caballi*, hematologic ratios, inflammatory response, piroplasmosis diagnosis, *Theileria equi*

## Abstract

Equine piroplasmosis (EP) is a tick-borne disease affecting equids worldwide. Diagnosis is based on direct methods (blood smear or PCR) or indirect methods (serology); however, these techniques yield positive and negative results. Thus, an alternative, reliable, rapid, and cost-effective complementary tool, such as hematologic parameters and newly described hematologic ratios, could help in the EP diagnosis. This study describes the changes induced by piroplasmosis infection on hematologic parameters and ratios in horses. *B. caballi* PCR-positive horses exhibited anemia and thrombocytopenia, whereas *T. equi* PCR-positive horses showed anemia and leukocytosis. *T. equi*-seropositive horses had lower neutrophil and lymphocyte percentages than seronegative horses. Hematologic ratios displayed significant differences between negative and infected horses (mostly those infected by *B. caballi*). Most of the variations observed in these ratios were those previously linked to the worst prognosis or outcome in horses and humans. This study also evaluates the ability to predict the EP status of these hematologic parameters and ratios, with platelet-related parameters showing a fair ability to predict a *B. caballi* PCR-positive result. While molecular methods remain the gold standard, complete hematology analysis and ratios could provide valuable complementary information to enhance the diagnostic accuracy of piroplasmosis in horses.

## Simple Summary

Equine piroplasmosis (EP) is a tick-borne parasitic disease affecting equids with important health and economic impacts worldwide. Diagnosis is based on direct detection of the causative agents (*Babesia caballi*, *Theileria equi*, *Theileria haneyi*) in the bloodstream (PCR or blood smear) or an indirect demonstration of the immune response of the host against these parasites (serology). However, it is unknown if other simpler and faster techniques (such as hematology) could help clinicians to suspect or discard this disease. In this study we describe hematologic differences between non-infected and EP-infected horses and evaluate the ability of these hematologic parameters and ratios to predict EP status.

## 1. Introduction

Equine piroplasmosis (EP) is a tick-borne disease caused by *Theileria equi*, *Theileria haneyi*, and *Babesia caballi* with a significant impact on equine health worldwide [1]. EP has serious economic consequences for the equine industry because of mortality, abortions, and reduced performance in affected horses [2]. Additionally, EP status can lead to restrictions on international trade and limit participation in global equine events [3].

Clinically, EP signs are highly variable, ranging from sudden death in hyperacute forms to mild, unspecific signs such as malaise, weight loss, and poor performance in chronic forms, or even coursing without evident clinical signs in asymptomatic carrier horses in endemic regions [4,5]. Moreover, infections caused by *B. caballi*, *T. equi*, and *T. haneyi* have similar clinical signs [6], which can overlap with other pathologies (piro-like diseases) such as anaplasmosis or leptospirosis [2]. Therefore, clinical signs alone are not adequate to diagnose this disease, mainly in those horses with asymptomatic infections (chronic carriers) [2].

Currently, EP diagnosis is based on direct detection of the parasite, either by microscopic examination of stained blood smears or by molecular methods (PCR), or indirect methods (serology) such as a competitive enzyme-linked immunosorbent assay (cELISA), complement fixation test (CFT), or indirect fluorescent antibody test (IFAT) [1,7,8]. Despite these tests being considered as gold standards for EP diagnosis (and thus needed for a final diagnosis), sensitivity and specificity are variable in each of these techniques, and false positive or negative results are possible in all of them [2]. Thus, it is clinically interesting to determine if any alternative, cost-effective, rapid, easy, and commonly performed technique (such as hematology) could aid in this diagnosis (i.e., helping to suspect EP in a screening health check or aiding in the differential between parasites). This could be particularly interesting in asymptomatic animals or animals in endemic areas where clinical signs are unspecific.

Although previous studies have evaluated the utility of hematological parameters in the diagnosis of piroplasmosis in infected horses [9,10,11], several methodological limitations can be raised from these studies. Briefly, hemograms were performed using basic impedance analyzers, leukocyte differentials relied on manual counts using light microscopy and blood smears, they lacked a detailed assessment of platelets, or EP diagnosis was based only on direct visualization of parasites in stained blood smears. Moreover, there was a marked bias in these studies due to the inclusion of only asymptomatic horses, only those with acute EP clinical signs, samples being collected from horses living in a small geographic region, or no distinction between infections caused by *T. equi* or *B. caballi* being noted. In this sense, a study reporting the relations between complete blood analysis (using newly available automatized cytometry-based analyzers) in a large population of horses in endemic areas with their molecular and serological results could better determine whether hematology can act as a rapid and cost-effective complementary tool in EP diagnosis.

Hematologic ratios are emerging biomarkers in human medicine linked to immune activation and neuroendocrine stress, which are able to reliably predict neurologic disease, cancer, disease severity, or mortality [12,13,14,15,16]. There is scarce information about these ratios in veterinary medicine, with recent studies describing their prognostic value in septic foals [17,18,19]. Since it has been reported that, depending on the clinical form, EP infection triggers a systemic inflammatory response [20], these biomarkers could be reliable and helpful complementary tools in EP diagnosis. However, to the authors’ knowledge, there is no current information on the utility of these ratios in the diagnosis of EP.

In this sense, we hypothesized that EP infection could induce changes in common hematologic parameters and novel hematologic ratios depending on clinical presentation and severity of the disease. Thus, the aims of this study were (a) to characterize the hematologic changes induced by the infection with *B. caballi* or *T. equi* in horses according to the diagnostic method; (b) to study the utility of hematologic ratios as biomarkers of EP infection; and (c) to evaluate the reliability of hematologic parameters and ratios to predict *B. caballi* or *T. equi* infections in horses.

## 2. Materials and Methods

### 2.1. Animals Inclusion Criteria and Study Design

Animals were retrospectively selected from a database of blood samples submitted to a national private veterinary reference laboratory (Gasset Laboratory, DAV Salud Group SL, Granada, Spain) for routine analysis during a three-year period (from January 2022 to December 2024). The minimum number of blood samples (1066) was calculated using the formula for a finite population, taking into consideration the number of horses registered in Spain in 2022 (722,158) [21], a 95% confidence interval (95% CI), and 3% maximum error.

The inclusion criteria were a complete hemogram together with a method of piroplasmosis diagnosis for both parasites, either a real-time qPCR or a serological technique (cELISA or IFAT). No information on clinical signs was included in the statistical analysis in order to avoid any bias. However, signalment information such as breed, sex, and age was retrieved.

Blood samples were shipped overnight. Samples with lipemia, hemolysis, or any abnormal sample processing (e.g., long storage, shipping delay longer than 24 h, clotted sample, erroneous tube) were discarded. No repeated analyses from the same horse were included. Blood samples from horses co-infected with both parasites were discarded, as well as blood samples from donkeys and mules.

In order to evaluate the effect of infection with *B. caballi* or *T. equi* on hematologic parameters and ratios, horses were grouped according to the diagnostic method used. Regarding PCR, horses were grouped in the following groups: non-EP-infected (T− B−), *B. caballi*-positive but *T. equi*-negative (B+ T−), and *B. caballi*-negative but *T. equi*-positive (B− T+). Concerning serology, horses were grouped as seronegative for both parasites (sB− sT−), *B. caballi*-seropositive but *T. equi*-seronegative (sB+ sT−), and *B. caballi*-seronegative but *T. equi*-seropositive (sB− sT+).

### 2.2. Hematologic Analysis

Blood samples collected in EDTA-containing tubes were analyzed using an automatized laser cytometry-based analyzer (Sysmex XN-1000V analyzer, Sysmex Corporation, Kobe, Japan). An internal quality assessment was performed weekly using two levels of the manufacturer’s quality control material (XNCHECK Level 1 and Level 2; Sysmex Corporation, Kobe, Japan).

The following red cell parameters were retrieved: red blood cell concentration (RBC), hematocrit (HCT), hemoglobin (Hb), mean corpuscular volume (MCV), mean corpuscular hemoglobin concentration (MCHC), mean corpuscular hemoglobin (MCH), and red cell distribution width (RDW).

Regarding leukocytes, the following parameters were included: white blood cell concentration (WBC), neutrophil concentration (NEU), lymphocyte concentration (LYM), monocyte concentration (MONO), eosinophil concentration (EOS), and basophil concentration (BASO), as well as the percentages of each leukocyte population (NEU%, LYM%, MONO%, EOS%, and BASO%, respectively).

Platelet analysis included platelet count (PLT), plateletcrit (PCT), mean platelet volume (MPV), and platelet distribution width (PDW).

The following hematologic ratios were calculated: neutrophil to lymphocyte ratio (NLR), neutrophil to monocyte ratio (NMR), lymphocyte to monocyte ratio (LMR), monocyte to lymphocyte ratio (MLR), eosinophil to lymphocyte ratio (ELR), platelet to WBC ratio (PWR), platelet to neutrophil ratio (PNR), platelet to lymphocyte ratio (PLR), platelet to monocyte ratio (PMR), and red cell distribution width to platelet ratio (RDW:PLT) [13,14,22].

### 2.3. Indirect (Serological) Detection

#### 2.3.1. cELISA

Serum samples were analyzed within 48 h from submission using two WHOA-approved equine commercial cELISA kits (*T. equi* or *B. caballi*, VMRD Inc., Pullman, WA, USA) [23,24]. According to the manufacturer, these kits have a sensitivity of 95% and a specificity of 99.5% for *T. equi* and a sensitivity of 100% and a specificity of 100% for *B. caballi*. The assays were performed by the same operator following the manufacturer’s protocols. Optical densities were read at 630 nm (ChroMate 4300, Awareness Technology Inc., Palm City, FL, USA). A horse was considered positive, both for *T. equi* and *B. caballi*, when the inhibition percentage was higher than 40%.

#### 2.3.2. IFAT

Serum samples were analyzed for the presence of IgG against *T. equi* and *B. caballi* within 48 h from submission using two validated commercial equine kits (MegaFLUO^®^ *T. equi* or *B. caballi*, Megacor Diagnostik, Hörbranz, Austria) [25,26]. Briefly, serum samples were diluted from 1:80 to 1:280 in phosphate-buffered saline (PBS). Pre-fixed slides were first incubated with 20 µL of serum for 30 min at 37 °C, followed by three washes with PBS and a second 30 min incubation with 20 µL of FITC anti-horse IgG conjugate. After washing with PBS, the fluorescence was evaluated using a trinocular fluorescence microscope (Balea 8100, Beortek SA, Vizcaya, Spain). Positive and negative controls were included in each assay. The cut-off for seropositivity, for both parasites, was a fluorescent signal greater than 1:80. The exact sensitivity and specificity of this test are unknown, but this technique is considered a highly specific confirmatory test [25].

### 2.4. Direct Detection

DNA was extracted from a 200 µL EDTA blood sample using an automatized nucleic acid extraction system (MagNA Pure 24 Instruments, Roche Diagnostics, Barcelona, Spain) following the manufacturer’s instructions. Extracted DNA was frozen at −20 °C until real-time qPCR was performed.

A real-time PCR was performed using commercial DNA *T. equi* or *B. caballi* detection kits (genetic PCR solutions, Alicante, Spain) in an automatized thermocycler (LightCycler 480, Roche Diagnostics, Barcelona, Spain). These kits contain individual ready-to-use tubes containing all the components needed to perform the PCR (mastermix, primers, DNase/RNase-free water, etc.). Internal, negative, and positive controls were also provided by the manufacturer in the same kits. The PCR conditions, in a final reaction volume of 20 µL (5 µL of DNA) for both parasites, were an initial activation at 95 °C for 2 min, followed by 40 cycles of 5 s at 95 °C (denaturation), and a final step at 60 °C for 20 s (hybridization/extension).

### 2.5. Statistical Analysis

The Kolmogorov–Smirnov test was used to assess normality. Results are expressed as mean ± standard deviation (SD) or median and interquartile range (IQR, 25th–75th percentiles) according to the distribution. The Tukey’s Hinges test was used to calculate the median and percentiles. Groups were compared using a one-way ANOVA or the Kruskal–Wallis test according to normality.

Receiver operating characteristic (ROC) curve analysis was performed to assess the ability of hematological parameters to predict EP status based on the area under the curve (AUC), which was classified as excellent (with an AUC between 0.9 and 1.0), good (0.8–0.9), fair (0.7–0.8), poor (0.6–0.7), and fail (<0.6). The sensitivity and specificity for each parameter were determined using the cut-off value with a higher likelihood ratio to differentiate between positive and negative animals. Accuracy and positive and negative predictive values were calculated based on those sensitivities and specificities.

All analyses were conducted using specific statistical packages (IBM SPSS Statistics 27, IBM Corporation, Armonk, NY, USA; and GraphPad Prism 9, San Diego, CA, USA). Values with *p* < 0.05 were considered significant.

## 3. Results

A total of 1121 horse blood samples were submitted to our laboratory for piroplasmosis analysis during the study period, with 283 fulfilling the inclusion criteria (11.1% submitted in 2022, 46.5% in 2023, and 42.5% in 2024). The median age was 5 (4) years old (ranging from 1 to 26 years old), with 24% mares and 76% males. The most common breed in our study population was Andalusian (69.3%), followed by their crossbred (18.4%), Lusitano (4.5%), Spanish Sport Horse (CDE, 1.3%), KWPN (0.8%), Hispano-Breton (0.8%), Catalan Pyrenean Horse (0.5%), Arabian (0.5%), Anglo-Arabian (0.5%), and Hispano-Arabian (0.5%). Other breeds comprising < 0.5% of our population were Argentine Polo, Frisian, Westphalian, French Trotter, Poni crossbred, Burguete, Zangersheide, Belgian Warmblood, etc. Nine horses were excluded due to co-infection with both parasites.

### 3.1. Changes in Hematologic Parameters According to EP Status in Each Diagnostic Method

The hematological results for non-EP-infected horses (B− T−), *B. caballi* PCR-positive (B+ T−), and *T. equi* PCR-positive (B− T+) are compiled in Table 1. *B. caballi* PCR-positive horses (B+ T− horses) had lower RBC (*p* < 0.01), HTC (*p* < 0.01), Hb (*p* = 0.02), and RDW (*p* < 0.01), but higher MCHC (*p* = 0.02) and MCH (*p* = 0.02) than non-infected horses (B− T−, Table 1). Although B− T+ horses also had lower RBC than negative ones, no differences were observed between both infected groups for any red cell parameter (Table 1). B− T+ horses had significantly (*p* < 0.05) higher WBC than control and *B. caballi*-infected groups. In contrast, B+ T− horses had significantly (*p* < 0.05) higher MONO and MONO% than all the other groups (Table 1). In addition, these horses also had significantly (*p* < 0.05) lower PLT and PCT than the rest of the groups (Table 1).

The hematological results in horses seronegative for both parasites (sT− sB−), *B. caballi*-seropositive (sB+ sT−), and *T. equi*-seropositive (sB− sT+) are compiled in Table 2. No differences were observed for any red cell parameter between the control group and both seropositive groups. In contrast, sB− sT+ horses had significantly (*p* < 0.05) higher lymphocyte concentrations and percentages but significantly (*p* < 0.05) lower neutrophil concentrations compared to the control group (Table 2). No differences were found among groups for any platelet parameter.

### 3.2. Changes in Hematologic Ratios According to EP Status in Each Diagnostic Method

When hematologic ratios were compared between horses with different PCR statuses, *B. caballi* infection induced changes in several of these biomarkers (Table 3). Briefly, NMR (*p* = 0.03), LMR (*p* = 0.004), PWR (*p* = 0.03), PNR (*p* = 0.03), and PMR (*p* = 0.03) were statistically lower compared to negative horses (Table 3). In contrast, MLR (*p* = 0.004) and RDW:PLT (*p* = 0.004) were higher compared to this group. Moreover, horses infected with *B. caballi* had higher PMR (*p* = 0.05) but lower MLR (*p* = 0.05) and RDW:PLT (*p* = 0.002) than those infected with *T. equi* (Table 3). No differences were observed between horses infected with *T. equi* and PCR-negative horses.

Regarding serology, sB− sT+ had lower NLR (*p* = 0.01), MLR (*p* = 0.05), and PLR (*p* < 0.001), but higher LMR (*p* = 0.05), compared to the control group (Table 4). sB+ sT− horses also had lower (*p* = 0.03) NLR compared to the control group. No differences were found between both seropositive groups.

### 3.3. Predictive Values of Hematologic Parameters and Ratios for EP Status

Based on the AUC in the ROC analysis, the following parameters showed a fair ability to predict a positive PCR for *B. caballi*: PLT (AUC 0.77 [95% CI: 0.62–0.90], *p* < 0.01), PCT (AUC 0.79 [95% CI: 0.66–0.92], *p* < 0.01), PWR (AUC 0.76 [95% CI: 0.62–0.91], *p* < 0.01), PNR (AUC 0.74 [95% CI: 0.60–0.88], *p* < 0.01), PMR (AUC 0.73 [95% CI: 0.57–0.89], *p* < 0.01), and RDW:PLT (AUC 0.74 [95% CI: 0.61–0.9], *p* < 0.01) (Table S1). Analytical accuracy, sensitivity, and specificity for these ratios and parameters were moderate to good, with an overall high negative predictive value (Table S1). In contrast, no parameter was found to possess a good or fair ability to predict a PCR-positive result for *T. equi* (Table S2).

Any parameter was found to be good to predict a seropositive horse for *B. caballi* (Table S3). In contrast, PLR (AUC 0.74 [95% CI: 0.62–0.85], *p* < 0.01), NLR (AUC 0.70 [95% CI: 0.57–0.84], *p* < 0.01), NEU% (AUC 0.69 [95% CI: 0.57–0.83], *p* < 0.01), LYM (AUC 0.69 [95% CI: 0.55–0.84], *p* < 0.01), and LYM% (AUC 0.69 [95% CI: 0.57–0.83], *p* < 0.01) had fair ability to predict a seropositive animal to *T. equi* (Table S4). These ratios showed a high negative predictive value (Table S4).

## 4. Discussion

This study is the first one describing the reliability and predictive value of different hematological parameters and hematological ratios in the diagnosis of EP. Although the hematological changes induced by both *B. caballi* and *T. equi* have already been described in different clinical forms [10,11,27], this study was designed to evaluate them in a large cohort of horses, independent of their clinical signs.

*B. caballi*-infected (PCR-positive) horses showed lower RBC, higher monocyte concentrations, and thrombocytopenia compared to negative horses, as well as significant differences in platelet- and monocyte-related ratios. Platelet counts, plateletcrit, and platelet-related ratios showed a fair ability to predict a positive PCR result for this parasite. In contrast, neither hematological parameters nor ratios were reliable to predict a positive serology for *B. caballi*, with this group only showing a lower NLR compared to seronegative horses. Regarding *T. equi* infection, lower RBC and higher WBC, but no thrombocytopenia, were observed compared to negative horses. In contrast, *T. equi* seropositivity induced a decrease in neutrophil concentrations and an increase in lymphocyte concentrations, as well as changes in NLR, LMR, PLR, and MLR. NLR and PLR showed a fair ability to predict a seropositive horse for *T. equi*.

Anemia is widely described in the EP literature [28], with *T. equi* infection usually causing a more severe disease [9,29]. In our study, PCR-positive horses, both for *B. caballi* and *T. equi*, presented lower RBC compared to negative animals, although this change was more marked in the former group (which also showed other erythrocytic variations typical of anemia, such as decreased HTC and Hb).

Red blood cell-related parameters were not changed in seropositive horses, neither against *B. caballi* nor *T. equi*. Similar findings (anemia in PCR-positive animals but lack of variations in seropositive horses) have been reported [10]. However, other authors have found lower hematocrits (without changes in RBC) [9] or lower RBC (without changes in HTC) [27] in seropositive horses. Noteworthy, no distinction between both parasites was performed in these studies.

Discrepancies in the presence of anemia between techniques could be explained by PCR being the preferred diagnostic method in acute forms in endemic regions, whereas serology is normally chosen in chronic asymptomatic horses (carrier form) or during pre-exportation screening (without clinical signs). While hemolysis (anemia, jaundice, etc.) is frequently observed in acute EP cases [10,30], asymptomatic carriers often lack any red blood cell abnormality [11]. In our study, anamnesis and clinical signs were not recorded to avoid any bias in the analysis.

EP-infected horses have been described to present higher WBC compared to negative ones [11,27], although no attempt was made to independently evaluate the effect of each parasite in these studies. In our study, *T. equi* PCR-positive horses showed higher WBC compared to negative animals, as previously described [10,28,29]. This finding could be linked to this parasite’s ability to invade peripheral blood mononuclear cells [4,31]. However, *T. equi*-seropositive horses lacked any change in WBC, contrary to one report in clinically affected horses [10]. On the other hand, horses positive for *B. caballi* (either using PCR or serology) had similar WBC compared to negative animals, as previously reported [9,10,30].

Regarding leukocyte differential counts, previous studies only reported granulocytes and lymphocytes or total white cell counts, not including a complete description of changes induced by EP on white cell differential counts [10,11,27]. In our study, *B. caballi* PCR-positive horses had higher monocyte concentrations and percentages than the other groups, but no change was noted in *B. caballi*-seropositive horses. In contrast, *T. equi*-seropositive horses had higher lymphocyte concentrations and percentages and lower neutrophil percentages compared to the control group. These differences could be linked to differences in clinical forms (acute versus chronic/asymptomatic) and differences in the type of immune response triggered by each parasite (adaptive versus innate) [32].

There are no studies available describing the effect of each parasite in the complete leukocyte differential. Previous studies have reported lower neutrophil (or granulocyte) percentages and higher lymphocyte percentages in PCR-positive horses compared to the control group, without changes in seropositive horses [11,27]. Contrary to our results, no changes in monocyte concentrations were observed in clinical or subclinical cELISA seropositive horses [30].

*B. caballi* PCR-positive horses had thrombocytopenia compared to the control group. This finding was not observed in horses PCR-positive to *T. equi*. Moreover, no changes in platelet concentrations were observed in seropositive (any parasite) horses. Our findings are similar to those in a previous report [10]. However, platelet count remains a controversial topic in EP, with some authors reporting normal concentrations in PCR- and ELISA-positive horses [11], other authors describing thrombocytopenia in both techniques [27], and even for both parasites [9].

No studies evaluating further platelet parameters in EP are available. In our study, *B. caballi* PCR-positive horses presented significantly lower plateletcrit than the rest of the groups, which further signifies the overall low platelet mass in this group. No differences were observed in MPV between groups.

Discrepancies between our study and previous ones evaluating hematologic parameters could be attributed to dissimilarities in the analyzers used in each study. To the authors’ knowledge, this is the first work using a new generation cytometry-based analyzer (XN-1000V, Sysmex) in piroplasmosis-infected horses. All previous studies used more imprecise methods such as analyzers based on impedance or older-generation laser techniques and manual techniques such as visualization of blood smears to calculate leukocyte differential counts [9,10,11,27,28,30]. In addition, other factors such as animal selection based on the clinical presentation (clinical, subclinical, or carrier horses) or those attributed to blood sample processing (i.e., anticoagulant, platelet aggregation, time until measurement, etc.) cannot be discarded.

Hematologic ratios are emerging biomarkers widely used in human medicine but have been only recently applied to equids. This study is the first one evaluating these novel markers in EP infection, as well as determining their predictive value in EP diagnosis. Among these ratios, only RDW:PLT and NLR have been previously studied in horses [17,18]. In our study, *B. caballi* PCR-positive horses had higher RDW:PLT than *T. equi* PCR positive and non-infected horses. It has been described that septic foals also showed higher RDW:PLT values compared to sick non-septic and healthy foals [17]. Consequently, RDW:PLT is currently considered an informative inflammatory indicator in septic horses, useful for risk prediction in these animals [19]. This ratio is used as a marker of the severity of inflammation in several human medicine pathologies, such as acute pancreatitis, myocardial disease, or breast tumors [33]. On the other hand, both *B. caballi-* and *T. equi*-seropositive horses presented a lower NLR compared to negative ones. Sick foals also have a lower NLR than healthy foals, with non-surviving foals displaying lower values [18]. In human medicine, low NLR has been linked with adrenal insufficiency or viral pathologies [34].

Although the rest of the ratios mentioned have not been specifically studied in horses, many of the variations observed in infected or seropositive animals in our study could be extrapolated to complications or the worst outcome in human medicine. For example, high LMR and low MLR (seen in *B. caballi*-infected horses and those seropositive against *T. equi*) are linked to the worst prognosis in patients with myocardial disease [22]; low PWR and PNR (*B. caballi* PCR-infected horses) are markers of the worst outcome in ischemic stroke patients [35,36]; and low PMR (*B. caballi* PCR-infected horses) can predict decompensated cirrhosis in hepatitis B-infected patients [37].

Since *B. caballi* PCR-positive horses displayed changes in monocyte concentrations and percentages and thrombocytopenia, all monocyte- and platelet-related ratios (mostly NMR and the platelet-related ratios such as PWR, PNR, PMR, and RDW:PLT) showed differences in comparison with the control group. Therefore, these ratios could contribute to different acute forms of babesiosis from acute theileriosis or chronic piroplasmosis infections. On the other hand, since *T. equi*-seropositive horses had higher lymphocyte and lower neutrophil concentrations, the ratios LMR, MLR, and PLR could be helpful tools to diagnose chronic forms of theileriosis. It is important to highlight that although differences in NMR, LMR, and MLR were observed for both parasites, the tendency was different depending on the causative agent, with an enhancement observed in *T. equi*-seropositive horses, whereas a diminution of these ratios was seen in *B. caballi* PCR-positive horses. Overall, *B. caballi* PCR-positive horses presented the most striking differences in these ratios compared with other groups (which was expected, since these horses also displayed many significant differences in hematological parameters). Whether this finding is related to a more severe disease in these animals or intrinsic differences in the pathological response to each parasite (and each clinical form) should be further investigated. Noteworthy, these ratios are commonly used in human medicine as outcome predictors, and their value as isolated diagnostic tools is not well characterized. Nonetheless, it is important to highlight that EP-infected horses should not be compared to septic foals or human patients with inflammatory disorders where these biomarkers have been described, and the interpretation of these biomarkers in EP-infected horses must be cautious. Further studies are needed to clarify the role of these biomarkers in the prognosis and outcome of EP-infected horses.

Our last objective was to study the ability of hematologic parameters and ratios to predict *B. caballi* or *T. equi* status in horses. It should be clarified that the final diagnosis of EP should always rely on PCR or serology tests, but any of these hematologic parameters or ratios (which are easy and cheap to determine) could help to discard (or suspect) EP infection; clinicians could prioritize further specific tests or avoid unnecessary ones. Although we found several significant differences between groups, a ROC curve study was determined to be the best method in order to ascertain the ability of each parameter and ratio to predict the EP status [15].

As expected, parameters and ratios more capable of predicting EP status were those where the most striking significant differences between groups were found. In the case of *B. caballi* PCR-positive horses, the presence of thrombocytopenia, low plateletcrit, high RDW:PLT, or low values in other platelet-related ratios (PWR, PNR, PMR) were deemed as fair predictors, with these findings showing high specificity and good to high sensitivity. Moreover, these abnormalities displayed a high predictive negative value, which means that any patient lacking them has a low probability of being PCR-positive. This could be extremely helpful for clinicians working in endemic areas in order to prioritize more specific EP tests in animals showing these disturbances and/or further investigate other piro-like diseases in horses lacking in them. Concerning serology, we only found fair predictors in horses seropositive to *T. equi*. In these animals, low neutrophils, PLR, NLR, and high lymphocytes showed good-to-fair specificities and predictive negative values (again, animals lacking these changes could be considered as being more probably negative for this test). Since we did not find any parameter able to predict EP status for both parasites (or both techniques), the isolated use of hematology to discard/confirm EP could be very risky and should not be endorsed.

Finally, it is important to highlight some weaknesses and limitations of this study. First, since groups were created based on blood sample submissions to our laboratory, group size is heterogeneous. Moreover, the number of animals infected with *B. caballi* or *T. equi* included in each group is not homogenous, since the prevalence for *T. equi* is higher in the whole country (Spain) than the one for *B. caballi*, independent of the region. Although blood smears were only performed in samples suspected of having platelet clumps, a systematic visualization of all submitted samples could have been of value in order to determine any cell morphological change induced by EP infection. In this sense, a citrated blood sample could have been better to evaluate platelet counts in order to discard pseudothrombocytopenia secondary to the use of EDTA.

## 5. Conclusions

*B. caballi* and *T. equi* infections induce significant changes in several hematologic parameters and ratios, mostly in platelets and leukocyte-related parameters. These hematologic parameters and ratios are alternative complementary tools that can be helpful for EP diagnosis, although they should be combined with a direct or indirect detection method depending on clinical presentation. Further techniques should be developed to allow rapid EP diagnosis or to evaluate response to the treatment.

## Figures and Tables

**Table 1:** Hematologic parameters (n = 159) in non-EP-infected horses (control group, n = 109), *B. caballi* PCR positives (B+ T−, n = 11), and *T. equi* PCR positives (B− T+, n = 39).

	RBC (×10^6^/μL)	HTC (%)	Hb (g/dL)	MCV (fL)	MCHC (g/dL)	MCH (pg)	RDW (%)	WBC (×10^3^/μL)	NEU (×10^3^/μL)	NEU (%)	LYM (×10^3^/μL)	LYM (%)	MONO (×10^3^/μL)	MONO (%)	EOS (×10^3^/μL)	EOS (%)	BASO (×10^3^/μL)	BASO (%)	PLT (×10^3^/μL)	PCT (%)	MPV (fL)	PDW (%)
**B− T−**	7.4 ± 0.1	34.2 ± 0.6	11.8 (3.0)	46.4 ± 0.4	35.1 (1.9)	16.3 (1.6)	21.7 (2.1)	7.7 (3.3)	4.8 (2.4)	62.1 ± 1.2	2.1 (1.5)	29.5± 1.2	0.4 (0.3)	4.5 (1.8)	0.1 (0.2)	1.5 (2.6)	0.05 (0.04)	0.5 (0.3)	112 (49)	0.09 (0.04)	8.4 (1.0)	12.9 (5.9)
**B+ T−**	5.7 ± 0.6 ^a^	26.8 ± 2.7 ^a^	10.2 (5.6) ^a^	47.2 ± 0.7	36.1 (1.8) ^a^	17.0 (1.0) ^a^	19.9 (4.5) ^a^	8.1 (3.8)	4.2 (1.9)	59.3 ± 3.5	2.3 (1.5)	28.1 ± 2.8	0.6 (0.7) ^a^	7.7 (8.7) ^a^	0.1 (0.3)	1.3 (4.1)	0.09 (0.08)	1.0 (1.6) ^a^	59 (39) ^a^	0.03 (0.05) ^a^	8.9 (2.3)	15.0 (6.8)
**B− T+**	6.7 ± 0.3 ^a^	31 ± 1.2	11.4 (3.3)	46.6 ± 0.7	35.2 (2.0)	16.7 (1.7)	21.3 (1.6) ^b^	9.1 (3.6) ^a^	5.2 (1.9)	58.5 ± 2.7	2.5 (1.7)	29.2 ± 2.0	0.4 (0.3)	5.1 (3.3) ^b^	0.2 (0.3)	2.0 (3.2)	0.04 (0.03)	0.5 (0.3) ^b^	116 (48) ^b^	0.1 (0.04) ^b^	8.6 (0.7)	10.3 (6.8)
**Reference** Range *	6–11	30–43	10.5–17.0	37–55	31.0–38.6	12.5–19.7	20.5–26.5	6–12	2.5–7.5	30–70	1.5–5.0	15–50	0.2–0.5	0–7	0–0.4	0–3	0–0.1	0–1	90–300	0.08–0.12	4.1–9.0	12.0–17.5

Data are expressed as median (IQR, interquartile range) or mean ± standard error (SE) according to distribution. BASO, basophils; EOS, eosinophils; Hb, hemoglobin; HTC, hematocrit; LYM, lymphocytes; MCH, mean corpuscular hemoglobin; MCV, mean corpuscular volume; MCHC, mean corpuscular hemoglobin concentration; MONO, monocytes; MPV, mean platelet volume; NEU, neutrophils; PCT, plateletcrit; PDW, platelet distribution width; PLT, platelets; RBC, red blood cells; RD0W, red cell distribution width; WBC, white blood cells. ᵃ *p* < 0.05 versus the control group; ᵇ *p* < 0.05 versus B+ T−. * Reference ranges internally established in our laboratory.

**Table 2:** Hematologic parameters (n = 124) in horses seronegative for both parasites (control group [sT− sB−], n = 96), *B. caballi*-seropositive (sB+ sT−, n = 8), and *T. equi*-seropositive (sB− sT+, n = 20).

	RBC (×10^6^/μL)	HTC (%)	Hb (g/dL)	MCV (fL)	MCHC (g/dL)	MCH (pg)	RDW (%)	WBC (×10^3^/μL)	NEU (×10^3^/μL)	NEU (%)	LYM (×10^3^/μL)	LYM (%)	MONO (×10^3^/μL)	MONO (%)	EOS (×10^3^/μL)	EOS (%)	BASO (×10^3^/μL)	BASO (%)	PLT (×10^3^/μL)	PCT (%)	MPV (fL)	PDW (%)
**sB− sT−**	7.1 ± 0.1	33.2 ± 0.6	11.7 (2.3)	47.3 ± 0.4	34.6 (1.7)	16.3 (1.7)	21.5 (1.9)	7.9 (3.5)	5.0 (3.1)	63.1 ± 1.2	2.1 (1.1)	28.3 ± 1.1	0.3 (0.2)	4.6 (2.1)	0.1 (0.2)	1.8 (2.7)	0.04 (0.04)	0.5 (0.5)	123 (57)	0.1 (0.05)	8.4 (0.9)	12.7 (6.1)
**sB+ sT−**	6.9 ± 0.3	32.7 ± 1.5	11.3 (1.6)	47.1 ± 1.1	34.3 (2.1)	16.2 (0.7)	21.5 (1.1)	7.6 (1.7)	3.9 (1.7)	55.8 ± 4.7	2.8 (0.5)	35.4 ± 4.0	0.3 (0.1)	4.6 (1.9)	0.2 (0.2)	2.8 (2.7)	0.04 (0.04)	0.5 (0.4)	112 (85)	0.1 (0.05)	8.3 (1.2)	11.2 (6.2)
**sB− sT+**	7.2 ± 0.4	34.1 ± 1.6	11.8 (2.9)	46.6 ± 0.8	34.8 (2.2)	16.1 (2.3)	22.3 (1.8) ^a^	8.1 (3.6)	4.6 (2.9)	54.2 ± 3.3 ^a^	2.9 (2.3) ^a^	37.5 ± 3.2 ^a^	0.4 (0.2)	4.6 (1.4)	0.1 (0.2)	2.1 (3.1)	0.04 (0.03)	0.5 (0.5)	95 (60)	0.08 (0.05)	8.7 (1.7)	12.3 (5.4)
**Reference** Range *	6–11	30–43	10.5–17.0	37–55	31.0–38.6	12.5–19.7	20.5–26.5	6–12	2.5–7.5	30–70	1.5–5.0	15–50	0.2–0.5	0–7	0–0.4	0–3	0–0.1	0–1	90–300	0.08–0.12	4.1–9.0	12.0–17.5

Data are expressed as median (IQR, interquartile range) or mean ± standard error (SE) according to distribution. BASO, basophils; EOS, eosinophils; Hb, hemoglobin; HTC, hematocrit; LYM, lymphocytes; MCH, mean corpuscular hemoglobin; MCV, mean corpuscular volume; MCHC, mean corpuscular hemoglobin concentration; MONO, monocytes; MPV, mean platelet volume; NEU, neutrophils; PCT, plateletcrit; PDW, platelet distribution width; PLT, platelets; RBC, red blood cells; RDW, red cell distribution width; WBC, white blood cells. ^a^ *p* < 0.05 versus the control group (sB− sT−). * Reference ranges internally established in our laboratory.

**Table 3:** Hematologic biomarkers (n = 159) in non-EP-infected horses (control group, n = 109), *B. caballi* PCR positives (B+ T−, n = 11), and *T. equi* PCR positives (B− T+, n = 39).

	NLR	NMR	LMR	MLR	ELR	PWR	PNR	PLR	PMR	RDW:PLT
**B− T−**	2.1 (2.1)	13.5 (7.0)	6.3 (4.4)	0.1 (0.1)	0.05 (0.1)	13.7 (8.6)	22.6 (16.1)	50.4 (42.1)	302 (210)	0.2 (0.1)
**B+ T−**	2.5 (2.3)	6.6 (7.1) ^a^	3.8 (3.2) ^a^	0.3 (0.3) ^a^	0.05 (0.1)	6.7 (8.5) ^a^	10.5 (14.3) ^a^	26.1 (35.8)	74 (205) ^a^	0.3 (0.3) ^a^
**B− T+**	1.9 (1.6)	11.8 (8.7)	6.2 (12.5)	0.1 (0.2) ^b^	0.07 (0.1)	12.3 (8.2)	22.5 (12.9)	42.0 (37.4)	273 (275) ^b^	0.2 (0.1) ^b^

Data are expressed as median (IQR, interquartile range) or mean ± standard error (SE) according to distribution. ELR, eosinophil to lymphocyte ratio; LMR, lymphocyte to monocyte ratio; MLR, monocyte to lymphocyte ratio; NLR, neutrophil to lymphocyte ratio; NMR, neutrophil to monocyte ratio; PLR, platelet to lymphocyte ratio; PMR, platelet to monocyte ratio; PNR, platelet to neutrophil ratio; PWR, platelet to WBC ratio; RDW:PLT, RDW to platelet ratio; WBC, white blood cells. ᵃ *p* < 0.05 versus the control group; ᵇ *p* < 0.05 versus B+ T−.

**Table 4:** Hematologic biomarkers (n = 124) in horses seronegative for both parasites (control group [sT− sB−], n = 96), *B. caballi*-seropositive (sB+ sT−, n = 8), or *T. equi*-seropositive (sB− sT+, n = 20).

	NLR	NMR	LMR	MLR	ELR	PWR	PNR	PLR	PMR	RDW:PLT
**sB− sT−**	2.1 (1.8)	12.9 (7.5)	6.4 (3.5)	0.1 (0.1)	0.1 (0.1)	14.7 (10.7)	24.5 (17.5)	55.8 (40.3)	311 (238)	0.2 (0.1)
**sB+ sT−**	1.3 (0.7) ^a^	10.4 (5.2)	7.6 (4.4)	0.1 (0.1)	0.1 (0.1)	14.1 (12.3)	29.8 (25.5)	43.5 (55.2)	325 (247)	0.2 (0.2)
**sB− sT+**	1.3 (1.0) ^a^	11.9 (7.8)	8.3 (3.7) ^a^	0.1 (0.1) ^a^	0.1 (0.1)	12.8 (7.4)	24.4 (18.4)	32.8 (20.9) ^a^	236 (159)	0.2 (0.1)

Data are expressed as median (IQR, interquartile range) or mean ± standard error (SE) according to distribution. ELR, eosinophil to lymphocyte ratio; LMR, lymphocyte to monocyte ratio; MLR, monocyte to lymphocyte ratio; NLR, neutrophil to lymphocyte ratio; NMR, neutrophil to monocyte ratio; PLR, platelet to lymphocyte ratio; PMR, platelet to monocyte ratio; PNR, platelet to neutrophil ratio; PWR, platelet to WBC ratio; RDW:PLT, RDW to platelet ratio; WBC, white blood cells. ᵃ *p* < 0.05 versus the control group (sB− T−).

## Data Availability

Data are available upon request to the corresponding author.

## Supplementary Materials

The following supporting information can be downloaded at: https://www.mdpi.com/article/10.3390/ani15101485/s1. Performance of hematologic parameters and ratios for predicting *B. caballi* infection by PCR (Table S1); *T. equi* infection by PCR (Table S2); *B. caballi* infection by serology (Table S3); and *T. equi* infection by serology (Table S4).
