# Supplementary material for: Assessment of Common Hematologic Parameters and Novel Hematologic Ratios for Predicting Piroplasmosis Infection in Horses
Source: Animals (Basel). 2025 May 20;15(10):1485. doi: 10.3390/ani15101485 (PMC12108503; doi:10.3390/ani15101485)
Supplement: Supplementary file 1 [file animals-15-01485-s001.zip › animals-3639700-supplementary/Table S1. PCR NEG VS PCR B+ J2.pdf]

Table S1. Performance of hematologic parameters and ratios for predicting *B. caballi* infection by PCR.

Variable	AUC (95% CI)	p value	SEN (95% CI)	SPE (95% CI)	ACC	PPV	NPV
**RBC**	0.641 (0.474-0.806)	0.058	0.389 (0.203-0.613)	0.962 (0.906-0.985)	0.876	0.636	0.900
**HTC**	0.632 (0.464-0.799)	0.075	0.389 (0.203-0.613)	0.981 (0.933-0.996)	0.893	0.778	0.902
**Hb**	0.635 (0.476-0.792)	0.069	0.389 (0.203-0.613)	0.962 (0.906-0.985)	0.876	0.636	0.900
**MCV**	0.505 (0.381-0.627)	0.949	1.000 (0.824-1.00)	0.229 (0.158-0.317)	0.331	0.182	1.000
**MCHC**	0.582 (0.426-0.737)	0.267	0.722 (0.491-0.875)	0.495 (0.401-0.589)	0.504	0.191	0.906
**MCH**	0.539 (0.390-0.687)	0.599	0.444 (0.245-0.662)	0.705 (0.611-0.783)	0.645	0.195	0.875
**RDW**	0.649 (0.479-0.817)	0.044	0.556 (0.337-0.754)	0.819 (0.734-0.881)	0.777	0.345	0.913
**WBC**	0.589 (0.431-0.746)	0.229	0.389 (0.203-0.613)	0.848 (0.766-0.904)	0.777	0.304	0.888
**NEU**	0.530 (0.388-0.672)	0.681	0.556 (0.337-0.754)	0.571 (0.475-0.661)	0.562	0.182	0.879
**NEU%**	0.605 (0.450-0.760)	0.155	0.556 (0.337-0.754)	0.743 (0.651-0.816)	0.711	0.270	0.905
**LYM**	0.562 (0.397-0.725)	0.405	0.500 (0.290-0.709)	0.705 (0.611-0.783)	0.669	0.225	0.889
**LYM%**	0.535 (0.377-0.691)	0.639	0.278 (0.125-0.508)	0.914 (0.845-0.954)	0.818	0.357	0.879
**MONO**	0.664 (0.511-0.817)	0.026	0.500 (0.290-0.709)	0.876 (0.799-0.926)	0.818	0.409	0.909
**MONO%**	0.640 (0.476-0.804)	0.058	0.500 (0.290-0.709)	0.876 (0.799-0.926)	0.818	0.409	0.909
**EOS**	0.501 (0.560-0.646)	0.989	0.278 (0.125-0.508)	0.829 (0.745-0.888)	0.744	0.217	0.867
**EOS%**	0.534 (0.392-0.675)	0.647	0.667 (0.437-0.837)	0.467 (0.374-0.561)	0.504	0.182	0.891
**BASO**	0.571 (0.397-0.744)	0.338	0.444 (0.245-0.662)	0.848 (0.766-0.904)	0.769	0.308	0.895
**BASO%**	0.674 (0.509-0.838)	0.019	0.611 (0.386-0.796)	0.771 (0.682-0.841)	0.702	0.275	0.914
**PLT**	0.763 (0.624-0.901)	0.000	0.833 (0.607-0.941)	0.760 (0.669-0.831)	0.760	0.359	0.951
**PCT**	0.794 (0.661-0.925)	<0.001	0.778 (0.547-0.910)	0.712 (0.618-0.789)	0.736	0.325	0.938
**MPV**	0.593 (0.444-0.741)	0.209	0.278 (0.125-0.508)	0.923 (0.855-0.960)	0.802	0.313	0.876
**PDW**	0.598 (0.452-0.742)	0.245	0.929 (0.685-0.996)	0.350 (0.254-0.459)	0.512	0.194	0.907
**NLR**	0.554 (0.396-0.711)	0.466	0.333 (0.162-0.562)	0.867 (0.788-0.918)	0.777	0.263	0.873
**NMR**	0.660 (0.493-0.825)	0.031	0.500 (0.290-0.709)	0.886 (0.810-0.933)	0.826	0.429	0.910
**LMR**	0.640 (0.489-0.789)	0.050	0.667 (0.437-0.837)	0.667 (0.572-0.749)	0.661	0.255	0.919
**MLR**	0.637 (0.487-0.786)	0.064	0.611 (0.386-0.796)	0.714 (0.621-0.791)	0.678	0.256	0.910
**ELR**	0.530 (0.382-0.676)	0.689	0.333 (0.162-0.562)	0.810 (0.724-0.873)	0.769	0.361	0.941
**PWR**	0.763 (0.620-0.906)	0.000	0.667 (0.437-0.837)	0.885 (0.809-0.932)	0.851	0.500	0.938
**PNR**	0.741 (0.604-0.877)	0.001	0.722 (0.491-0.875)	0.817 (0.744-0.882)	0.802	0.406	0.944
**PLR**	0.713 (0.566-0.859)	0.004	0.556 (0.337-0.754)	0.865 (0.786-0.918)	0.818	0.417	0.918
**PMR**	0.729 (0.570-0.887)	0.002	0.667 (0.437-0.837)	0.827 (0.742-0.887)	0.802	0.400	0.934
**RDW:PLT**	0.745 (0.608-0.88)	0.001	0.667 (0.437-0.837)	0.837 (0.753-0.895)	0.802	0.400	0.934

ACC, accuracy; AUC, area under curve; BASO, basophils; CI, confidence interval; ELR, eosinophil to lymphocyte ratio; EOS, eosinophils; Hb, hemoglobin; HTC, hematocrit; LMR, lymphocyte to monocyte ratio; LYM, lymphocytes; MCH, mean corpuscular hemoglobin; MCV, mean corpuscular volume; MCHC, mean corpuscular hemoglobin concentration; MLR, monocyte to lymphocyte ratio; MONO, monocytes; MPV, mean platelet volume; NEU, neutrophils; NLR, neutrophil to lymphocyte ratio; NMR, neutrophil to monocyte ratio; NPV, negative predictive value; PCT, plateletcrit; PDW, platelet distribution width; PLR, platelet to lymphocyte ratio; PLT, platelets; PMR, Platelet to monocyte ratio; PNR, platelet to neutrophil ratio; PPV, positive predictive value; PWR, platelet to WBC ratio; RBC, red blood cells; RDW, red cell distribution width; RDW:PLT, RDW to platelet ratio; SEN, sensitivity; SPE, specificity; WBC, white blood cells.
