# Supplementary material for: Assessment of Common Hematologic Parameters and Novel Hematologic Ratios for Predicting Piroplasmosis Infection in Horses
Source: Animals (Basel). 2025 May 20;15(10):1485. doi: 10.3390/ani15101485 (PMC12108503; doi:10.3390/ani15101485)
Supplement: Supplementary file 1 [file animals-15-01485-s001.zip › animals-3639700-supplementary/Table S2. PCR NEG VS PCR T+.pdf]

Table S2. Performance of hematologic parameters and ratios for predicting *T. equi* infection by PCR.

Variable	AUC (95% CI)	p value	SEN (95% CI)	SPE (95% CI)	ACC	PPV	NPV
**RBC**	0.608 (0.506-0.709)	0.037	0.267 (0.159-0.410)	0.952 (0.893-0.979)	0.718	0.714	0.718
**HTC**	0.589 (0.486-0.690)	0.085	0.267 (0.159-0.410)	0.925 (0.858-0.961)	0.725	0.600	0.744
**Hb**	0.590 (0.487-0.692)	0.081	0.333 (0.213-0.479)	0.886 (0.810-0.933)	0.711	0.538	0.748
**MCV**	0.505 (0.408-0.602)	0.918	0.889 (0.765-0.951)	0.257 (0.183-0.348)	0.443	0.339	0.839
**MCHC**	0.504 (0.402-0.606)	0.933	0.356 (0.232-0.501)	0.705 (0.611-0.783)	0.584	0.327	0.710
**MCH**	0.511 (0.407-0.615)	0.828	0.422 (0.289-0.567)	0.705 (0.611 - 0.783)	0.604	0.365	0.732
**RDW**	0.559 (0.458-0.659)	0.252	0.689 (0.543-0.804)	0.476 (0.383-0.570)	0.537	0.357	0.769
**WBC**	0.639 (0.542-0.734)	0.007	0.756 (0.613-0.857)	0.524 (0.429-0.616)	0.591	0.405	0.831
**NEU**	0.581 (0.483-0.678)	0.116	0.667 (0.520-0.786)	0.571 (0.475-0.661)	0.597	0.400	0.797
**NEU%**	0.574 (0.471-0.676)	0.150	0.356 (0.232-0.501)	0.810 (0.724-0.873)	0.664	0.429	0.737
**LYM**	0.610 (0.507-0.711)	0.034	0.578 (0.433-0.710)	0.686 (0.591-0.766)	0.651	0.441	0.789
**LYM%**	0.524 (0.420-0.628)	0.640	0.222 (0.125-0.362)	0.876 (0.799-0.926)	0.678	0.435	0.722
**MONO**	0.606 (0.505-0.706)	0.040	0.844 (0.712-0.922)	0.343 (0.259-0.437)	0.483	0.352	0.829
**MONO%**	0.544 (0.431-0.656)	0.395	0.467 (0.329-0.609)	0.771 (0.682-0.841)	0.671	0.457	0.767
**EOS**	0.579 (0.481-0.676)	0.128	0.600 (0.454-0.729)	0.581 (0.485-0.670)	0.577	0.375	0.766
**EOS%**	0.547 (0.445-0.647)	0.368	0.267 (0.159-0.410)	0.838 (0.755-0.896)	0.658	0.400	0.723
**BASO**	0.525 (0.421-0.627)	0.633	0.133 (0.062-0.261)	0.943 (0.880-0.973)	0.698	0.500	0.709
**BASO%**	0.559 (0.454-0.664)	0.252	0.200 (0.109-0.338)	0.933 (0.868-0.967)	0.698	0.500	0.715
**PLT**	0.512 (0.408-0.615)	0.812	0.356 (0.232-0.501)	0.760 (0.669-0.831)	0.631	0.375	0.725
**PCT**	0.505 (0.401-0.609)	0.919	0.467 (0.329-0.609)	0.587 (0.490-0.676)	0.557	0.356	0.750
**MPV**	0.554 (0.454-0.653)	0.299	0.689 (0.543-0.804)	0.433 (0.341-0.528)	0.497	0.344	0.774
**PDW**	0.535 (0.422-0.647)	0.539	0.256 (0.145-0.410)	0.888 (0.799-0.939)	0.705	0.526	0.731
**NLR**	0.560 (0.456-0.662)	0.253	0.864 (0.732-0.936)	0.276 (0.199-0.368)	0.443	0.333	0.800
**NMR**	0.566 (0.457-0.674)	0.203	0.356 (0.232-0.501)	0.829 (0.745-0.888)	0.685	0.471	0.748
**LMR**	0.505 (0.396-0.612)	0.927	0.356 (0.232-0.501)	0.762 (0.672-0.833)	0.631	0.375	0.725
**MLR**	0.507 (0.399-0.614)	0.894	0.318 (0.200-0.465)	0.819 (0.734-0.881)	0.651	0.400	0.728
**ELR**	0.521 (0.419-0.621)	0.694	0.955 (0.848-0.991)	0.191 (0.126-0.276)	0.389	0.323	0.842
**PWR**	0.581 (0.479-0.682)	0.116	0.600 (0.454-0.729)	0.596 (0.500-0.685)	0.597	0.391	0.775
**PNR**	0.565 (0.464-0.665)	0.213	0.500 (0.358-0.641)	0.635 (0.539-0.722)	0.591	0.367	0.742
**PLR**	0.605 (0.505-0.703)	0.045	0.568 (0.422-0.703)	0.625 (0.529-0.712)	0.604	0.391	0.765
**PMR**	0.586 (0.481-0.689)	0.097	0.422 (0.289-0.567)	0.817 (0.732-0.879)	0.698	0.500	0.766
**RDW:PLT**	0.506 (0.402-0.609)	0.910	0.600 (0.454-0.729)	0.539 (0.443-0.631)	0.544	0.351	0.750

ACC, accuracy; AUC, area under curve; BASO, basophils; CI, confidence interval; ELR, eosinophil to lymphocyte ratio; EOS, eosinophils; Hb, hemoglobin; HTC, hematocrit; LMR, lymphocyte to monocyte ratio; LYM, lymphocytes; MCH, mean corpuscular hemoglobin; MCV, mean corpuscular volume; MCHC, mean corpuscular hemoglobin concentration; MLR, monocyte to lymphocyte ratio; MONO, monocytes; MPV, mean platelet volume; NEU, neutrophils; NLR, neutrophil to lymphocyte ratio; NMR, neutrophil to monocyte ratio; NPV, negative predictive value; PCT, plateletcrit; PDW, platelet distribution width; PLR, platelet to lymphocyte ratio; PLT, platelets; PMR, Platelet to monocyte ratio; PNR, platelet to neutrophil ratio; PPV, positive predictive value; PWR, platelet to WBC ratio; RBC, red blood cells; RDW, red cell distribution width; RDW:PLT, RDW to platelet ratio; SEN, sensitivity; SPE, specificity; WBC, white blood cells.
