# Supplementary material for: Assessment of Common Hematologic Parameters and Novel Hematologic Ratios for Predicting Piroplasmosis Infection in Horses
Source: Animals (Basel). 2025 May 20;15(10):1485. doi: 10.3390/ani15101485 (PMC12108503; doi:10.3390/ani15101485)
Supplement: Supplementary file 1 [file animals-15-01485-s001.zip › animals-3639700-supplementary/Table S3. Sero NEG VS Sero B+.pdf]

Table S3. Performance of hematologic parameters and ratios for predicting *B. caballi* infection by serology.

Variable	AUC (95% CI)	p value	SEN (95% CI)	SPE (95% CI)	ACC	PPV	NPV
RBC	0.563 (0.40-0.723)	0.900	0.516 (0.595-0.994)	0.315 (0.227-0.417)	0.374	0.118	0.935
HTC	0.579 (0.418-0.744)	0.900	0.413 (0.595-0.994)	0.326 (0.237-0.428)	0.384	0.130	0.967
Hb	0.612 (0.461-0.763)	0.800	0.246 (0.490-0.964)	0.551 (0.447-0.649)	0.626	0.186	0.964
MCV	0.582 (0.409-0.754)	0.800	0.397 (0.490-0.964)	0.438 (0.339-0.541)	0.465	0.123	0.929
MCHC	0.530 (0.344-0.715)	0.700	0.758 (0.396-0.892)	0.607 (0.502-0.701)	0.596	0.125	0.915
MCH	0.578 (0.424-0.730)	0.900	0.423 (0.595-0.994)	0.461 (0.360-0.563)	0.525	0.164	0.977
RDW	0.571 (0.432-0.708)	0.800	0.465 (0.490-0.964)	0.483 (0.382-0.585)	0.475	0.138	0.951
WBC	0.548 (0.382-0.714)	0.900	0.618 (0.595-0.994)	0.315 (0.227-0.417)	0.374	0.129	0.966
NEU	0.569 (0.370-0.766)	0.800	0.479 (0.490-0.964)	0.494 (0.392-0.596)	0.525	0.151	0.957
NEU%	0.693 (0.508-0.878)	0.700	0.046 (0.396-0.892)	0.730 (0.630-0.811)	0.717	0.200	0.942
LYM	0.744 (0.593-0.894)	0.900	0.012 (0.595-0.994)	0.652 (0.548-0.742)	0.677	0.225	0.983
LYM%	0.698 (0.512-0.883)	0.600	0.041 (0.312-0.831)	0.787 (0.690-0.858)	0.768	0.240	0.946
MONO	0.570 (0.409-0.730)	1.000	0.468 (0.722-1.000)	0.247 (0.169-0.346)	0.313	0.128	1.000
MONO%	0.507 (0.313-0.700)	0.900	0.945 (0.595-0.994)	0.202 (0.131-0.297)	0.253	0.100	0.895
EOS	0.599 (0.446-0.752)	0.700	0.304 (0.396-0.892)	0.539 (0.436-0.639)	0.545	0.143	0.940
EOS%	0.582 (0.416-0.748)	1.000	0.397 (0.722-1.000)	0.191 (0.122-0.284)	0.212	0.114	1.000
BASO	0.611 (0.458-0.764)	0.800	0.250 (0.490-0.964)	0.438 (0.339-0.541)	0.455	0.133	0.949
BASO%	0.571 (0.430-0.711)	0.800	0.465 (0.490-0.964)	0.416 (0.318-0.519)	0.384	0.119	0.938
PLT	0.506 (0.311-0.701)	0.600	0.949 (0.312-0.831)	0.551 (0.447-0.649)	0.556	0.114	0.909
PCT	0.506 (0.327-0.684)	1.000	0.949 (0.722-1.000)	0.101 (0.054-0.181)	0.192	0.111	1.000
MPV	0.522 (0.339-0.704)	0.500	0.819 (0.236-0.763)	0.727 (0.626-0.809)	0.707	0.172	0.929
PDW	0.629 (0.436-0.821)	1.000	0.199 0.700-1.000)	0.289 0.215-0.375)	0.121	0.103	1.000
NLR	0.701 (0.517-0.885)	0.700	0.038 0.396-0.892)	0.697 0.594-0.782)	0.697	0.206	0.954
NMR	0.624 (0.424-0.822)	0.600	0.202 0.312-0.831)	0.764 0.666-0.840)	0.747	0.222	0.944
LMR	0.623 (0.441-0.803)	0.600	0.206 0.312-0.831)	0.697 0.594-0.782)	0.687	0.182	0.939
MLR	0.621 (0.437-0.805)	0.600	0.210 0.312-0.831)	0.685 0.583-0.772)	0.667	0.171	0.938
ELR	0.511 (0.372-0.649)	0.900	0.908 (0.595-0.994)	0.337 (0.247-0.440)	0.354	0.125	0.963
PWR	0.520 (0.324-0.715)	0.500	0.834 (0.236-0.763)	0.652 (0.548-0.742)	0.626	0.114	0.906
PNR	0.561 (0.374-0.746)	0.500	0.531 (0.236-0.763)	0.719 (0.618-0.801)	0.697	0.167	0.928
PLR	0.593 (0.394-0.791)	0.500	0.335 (0.236-0.763)	0.764 (0.666-0.840)	0.737	0.192	0.932
PMR	0.539 (0.352-0.726)	0.400	0.684 (0.168-0.687)	0.753 (0.654-0.830)	0.717	0.154	0.918
RDW:PLT	0.503 (0.316-0.690)	0.600	0.972 (0.312-0.831)	0.584 (0.480-0.681)	0.586	0.140	0.929

ACC, accuracy; AUC, area under curve; BASO, basophils; CI, confidence interval; ELR, eosinophil to lymphocyte ratio; EOS, eosinophils; Hb, hemoglobin; HTC, hematocrit; LMR, lymphocyte to monocyte ratio; LYM, lymphocytes; MCH, mean corpuscular hemoglobin; MCV, mean corpuscular volume; MCHC, mean corpuscular hemoglobin concentration; MLR, monocyte to lymphocyte ratio; MONO, monocytes; MPV, mean platelet volume; NEU, neutrophils; NLR, neutrophil to lymphocyte ratio; NMR, neutrophil to monocyte ratio; NPV, negative predictive value; PCT, plateletcrit; PDW, platelet distribution width; PLR, platelet to lymphocyte ratio; PLT, platelets; PMR, Platelet to monocyte ratio; PNR, platelet to neutrophil ratio; PPV, positive predictive value; PWR, platelet to WBC ratio; RBC, red blood cells; RDW, red cell distribution width; RDW:PLT, RDW to platelet ratio; SEN, sensitivity; SPE, specificity; WBC, white blood cells.
