# Supplementary material for: Assessment of Common Hematologic Parameters and Novel Hematologic Ratios for Predicting Piroplasmosis Infection in Horses
Source: Animals (Basel). 2025 May 20;15(10):1485. doi: 10.3390/ani15101485 (PMC12108503; doi:10.3390/ani15101485)
Supplement: Supplementary file 1 [file animals-15-01485-s001.zip › animals-3639700-supplementary/Table S4. Sero NEG VS Sero T+.pdf]

Table S4. Performance of hematologic parameters and ratios for predicting *T. equi* infection by serology.

Variable	AUC (95% CI)	p value	SEN (95% CI)	SPE (95% CI)	ACC	PPV	NPV
**RBC**	0.563 (0.409-0.716)	0.361	0.045 (0.023-0.218)	0.989 (0.939-0.999)	0.802	0.500	0.807
**HTC**	0.552 (0.406-0.697)	0.453	0.182 (0.073-0.385)	0.955 (0.890-0.982)	0.946	0.444	1.000
**Hb**	0.547 (0.399-0.694)	0.496	0.227 (0.101-0.434)	0.944 (0.875-0.975)	0.936	0.455	1.000
**MCV**	0.564 (0.430-0.698)	0.351	0.455 (0.269-0.653)	0.719 (0.618-0.801)	0.667	0.286	0.842
**MCHC**	0.535 (0.398-0.670)	0.618	0.045 (0.023-0.218)	0.989 (0.939-0.999)	0.793	0.333	0.806
**MCH**	0.558 (0.411-0.704)	0.401	0.227 (0.101-0.434)	0.944 (0.875-0.975)	0.793	0.444	0.824
**RDW**	0.665 (0.550-0.779)	0.017	0.727 (0.518-0.868)	0.618 (0.514-0.712)	0.631	0.314	0.900
**WBC**	0.544 (0.399-0.687)	0.527	0.091 (0.016-0.278)	0.989 (0.939-0.999)	0.811	0.667	0.815
**NEU**	0.555 (0.416-0.693)	0.429	0.227 (0.101-0.434)	0.955 (0.890-0.982)	0.811	0.556	0.833
**NEU%**	0.699 (0.566-0.832)	0.004	0.136 (0.047-0.333)	0.989 (0.939-0.999)	0.820	0.750	0.822
**LYM**	0.698 (0.552-0.844)	0.004	0.636 (0.429-0.802)	0.775 (0.678-0.849)	0.748	0.412	0.896
**LYM%**	0.699 (0.566-0.831)	0.004	0.136 (0.047-0.333)	0.989 (0.939-0.999)	0.820	0.750	0.822
**MONO**	0.548 (0.409-0.685)	0.492	0.546 (0.346-0.730)	0.663 (0.559-0.752)	0.622	0.273	0.851
**MONO%**	0.525 (0.395-0.655)	0.714	0.364 0.197-0.570)	0.708 (0.606-0.792)	0.649	0.242	0.821
**EOS**	0.514 (0.376 0.651)	0.836	0.409 (0.232-0.612)	0.730 (0.630-0.811)	0.667	0.273	0.833
**EOS%**	0.507 (0.372-0.641)	0.918	0.318 (0.163-0.526)	0.798 (0.702-0.868)	0.703	0.280	0.826
**BASO**	0.528 (0.393-0.663)	0.684	0.818 (0.614-0.926)	0.270 (0.188-0.370)	0.288	0.200	0.813
**BASO%**	0.502 (0.365-0.638)	0.979	0.091 (0.016-0.278)	0.966 (0.905-0.990)	0.793	0.400	0.811
**PLT**	0.587 (0.451-0.722)	0.209	0.591 (0.387-0.767)	0.607 (0.502-0.701)	0.604	0.271	0.857
**PCT**	0.518 (0.377-0.657)	0.799	0.455 (0.269-0.653)	0.685 (0.583-0.772)	0.640	0.250	0.827
**MPV**	0.550 (0.395-0.705)	0.466	0.409 (0.232-0.612)	0.807 (0.712-0.875)	0.721	0.333	0.845
**PDW**	0.547 (0.405-0.689)	0.518	0.833 (0.607-0.941)	0.298 (0.223-0.384)	0.171	0.150	0.364
**NLR**	0.704 (0.572-0.836)	0.003	0.682 (0.473-0.836)	0.674 (0.571-0.762)	0.667	0.326	0.882
**NMR**	0.580 (0.440-0.719)	0.246	0.409 (0.232-0.612)	0.843 (0.753-0.903)	0.757	0.391	0.852
**LMR**	0.657 (0.524-0.789)	0.023	0.500 (0.307-0.692)	0.798 (0.702-0.868)	0.739	0.379	0.866
**MLR**	0.653 (0.520-0.784)	0.027	0.500 (0.307-0.692)	0.787 (0.690-0.858)	0.730	0.367	0.864
**ELR**	0.553 (0.427-0.679)	0.440	0.636 (0.429-0.802)	0.551 0.447-0.649)	0.532	0.241	0.849
**PWR**	0.606 (0.483-0.729)	0.124	0.818 (0.614-0.926)	0.416 (0.318-0.519)	0.495	0.257	0.902
**PNR**	0.508 (0.376-0.639)	0.912	0.909 (0.721-0.983)	0.236 (0.159-0.333)	0.369	0.227	0.913
**PLR**	0.737 (0.625-0.849)	0.001	0.773 (0.565-0.898)	0.652 (0.548-0.742)	0.676	0.354	0.921
**PMR**	0.608 (0.476-0.740)	0.117	0.591 (0.387-0.767)	0.685 (0.583-0.772)	0.667	0.317	0.871
**RDW:PLT**	0.597 (0.463-0.730)	0.160	0.546 (0.346-0.730)	0.719 (0.618-0.801)	0.676	0.316	0.863

ACC, accuracy; AUC, area under curve; BASO, basophils; CI, confidence interval; ELR, eosinophil to lymphocyte ratio; EOS, eosinophils; Hb, hemoglobin; HTC, hematocrit; LMR, lymphocyte to monocyte ratio; LYM, lymphocytes; MCH, mean corpuscular hemoglobin; MCV, mean corpuscular volume; MCHC, mean corpuscular hemoglobin concentration; MLR, monocyte to lymphocyte ratio; MONO, monocytes; MPV, mean platelet volume; NEU, neutrophils; NLR, neutrophil to lymphocyte ratio; NMR, neutrophil to monocyte ratio; NPV, negative predictive value; PCT, plateletcrit; PDW, platelet distribution width; PLR, platelet to lymphocyte ratio; PLT, platelets; PMR, Platelet to monocyte ratio; PNR, platelet to neutrophil ratio; PPV, positive predictive value; PWR, platelet to WBC ratio; RBC, red blood cells; RDW, red cell distribution width; RDW:PLT, RDW to platelet ratio; SEN, sensitivity; SPE, specificity; WBC, white blood cells.
